# Study protocol: The clinical features, epidemiology, and causes of paediatric encephalitis in southern Vietnam

**DOI:** 10.12688/wellcomeopenres.16770.1

**Published:** 2021-05-28

**Authors:** Nguyen Hoang Thien Huong, Nguyen Duc Toan, Du Tuan Quy, Truong Huu Khanh, Le Quoc Thinh, Le Nguyen Thanh Nhan, Ngo Ngoc Quang Minh, Hugo Turner, Louise Thwaites, Sarosh Irani, Nguyen Thanh Hung, Le Van Tan

**Affiliations:** 1 Oxford University Clinical Research Unit, Ho Chi Minh City, 700000, Vietnam; 2 Children's Hospital 1, Ho Chi Minh City, 700000, Vietnam; 3 Nuffield Department of Clinical Neurosciences, University of Oxford, Oxford, UK

**Keywords:** encephalitis, next-generation sequencing, pathogens, autoimmune, anti-NMDAR

## Abstract

Encephalitis is a major cause of morbidity and mortality worldwide. The clinical syndrome of encephalitis consists of altered mental status, seizures, neurologic signs, and is often accompanied by fever, headache, nausea, and vomiting. The encephalitis in children has been known that more common than in adult, with the incidence rate of infants was 3.9 times higher than that of people 20-44 years of age. The reported incidence of hospitalization attributed to paediatric encephalitis ranged from 3 to 13 admissions per 100,000 children per year with the overall mortality ranging from 0 to 7%. There are however more than 100 pathogens that can cause encephalitis and accurate diagnosis is challenging. Over 50% of patients with encephalitis are left undiagnosed despite extensive laboratory investigations. Furthermore, recent studies in high-income settings have suggested autoimmune encephalitis has now surpassed infectious aetiologies, mainly due to increased awareness and diagnostic capacity, which further challenges routine diagnosis and clinical management, especially in developing countries.

There are limited contemporary data on the causes of encephalitis in children in Vietnam. Improving our knowledge of the causative agents of encephalitis in this resource-constrained setting remains critical to informing case management, resource distribution and vaccination strategy. Therefore, we conduct a prospective observational study to characterise the clinical, microbiological, and epidemiological features of encephalitis in a major children’s hospital in southern Vietnam. Admission clinical samples will be collected alongside meta clinical data and from each study participants. A combination of classical assays (serology and PCR) and metagenomic next-generation sequencing will used to identify the causative agents. Undiagnosed patients with clinical presentations compatible with autoimmune encephalitis will then be tested for common forms of the disease. Finally, using direct- and indirect costs, we will estimate the economic burden of hospitalization and seven days post hospital discharge of paediatric encephalitis in our setting.

## Introduction

Encephalitis is an inflammation of the brain parenchyma and is associated with neurologic dysfunction. The clinical features include (but are not limited to) altered mental status (decreased level of consciousness, lethargy, personality change, unusual behaviour), seizures, and/or focal neurologic signs, which are often accompanied by fever, headache, nausea, and vomiting ^1^. Encephalitis is a significant cause of morbidity and mortality worldwide ^2^, especially in the paediatric population ^3^. In a study of 7000 children admitted to hospital with suspected encephalitis in the United States between 2004 and 2013, 40% of children required admission to the paediatric intensive care unit, with a median length of hospital stay of 16 days ^4^. The overall mortality of childhood encephalitis ranges from 0 to 7% ^5, 6^. However, neurological deficits are recorded in the majority of the patients at hospital discharge. Persistent neurologic consequences may include personality change, behaviour disorder (including attention deficit disorder), movement disorder (including tic disorders), intellectual disability, learning disorders, blindness, paresis, ataxia, recurrent headaches, and sleeping problems ^7, 8^.

Clinical outcomes of encephalitis are highly dependent on the identification of the causative agents and timely initiation of appropriate clinical management approaches. Current diagnostics are inadequate and globally the causative agents are only identified in <50% of patients ^9^. In the California Encephalitis Project, 1570 patients who were at least six months of age (median age 23 years) were enrolled in the study between 1998 and 2005, and despite extensive microbiologic investigations, no aetiology was identified in 63% of cases ^10^. Of the patients with an aetiology identified, a confirmed or probable infectious aetiology was identified in 16%, with viruses (enteroviruses and herpes simplex virus) being the most common causes, accounting for 69% of the detected pathogens. The most commonly identified pathogenic viruses were enteroviruses (for 25% of cases) and herpes simplex virus-1 (for 24% of cases) ^11^. Results from studies conducted elsewhere in western countries showed that a confirmed or probable infectious aetiology was identified in approximately 40% – 60% of cases, and a possible cause was identified in approximately 25% ^6, 12, 13^. In southern Vietnam, there has been only one comprehensive study on paediatric encephalitis conducted from 2003 – 2004. Of 194 enrolled children presenting with encephalitis, a viral agent was established in 41%. The most common pathogen was Japanese encephalitis virus (n = 50, 26%), followed by enteroviruses (n = 18, 9.3%), dengue virus (n = 9, 4.6%), herpes simplex virus (n = 1), cytomegalovirus (n = 1) and influenza A virus (n = 1). An unfavourable outcome was recorded in 54% of the patients, including 57 (29%) fatal cases ^11^.

In addition, Vietnam and Asia are highly susceptible to emerging infectious diseases, including those caused by novel neurotropic pathogens (e.g., Nipah virus, enterovirus A71, and SARS-CoV-2). New diagnostic approaches are thus urgently required for the rapid response to these evolving challenges and to improve clinical outcomes.

The emergence of new pathogens causing encephalitis, the wide spectrum of known viruses responsible, the low sensitivity of current diagnostic methods and the high mortality and morbidity associated with encephalitis make it an attractive candidate for novel diagnostic assays such as metagenomic next-generation sequencing (mNGS). mNGS allows for nucleic acids to be directly sequenced from clinical samples without the requirement of prior knowledge of the targeted pathogens. As such, a mNGS-based diagnostic approach has the potential to revolutionize the study of genomics and the diagnosis of infectious diseases. 

Anti-NMDAR encephalitis was first discovered in 2007 as a phenomenon associated with underlying ovarian teratoma. Over the last years, its epidemiology has shifted substantially; it has more often been reported in female patients without tumour, males and children. Anti-NMDAR has since become one of the most common forms of encephalitis, especially in children, worldwide ^14– 19^. In Vietnam we have recently described the diagnosis, clinical management, and long-term outcome of the first case series of anti-NMDAR encephalitis admitted to the Hospital for Tropical Disease (HTD) between 2015 and 2016 ^20^. At Children’s Hospital 1 in Ho Chi Minh City, Vietnam, anti-NMDAR encephalitis has recently been diagnosed in 4/6 cases.

This study aims to improve our knowledge about the causes of paediatric encephalitis in southern Vietnam. Specifically, our aims are to describe the epidemiology, clinical profiles, in-hospital outcome and causes of encephalitis in children in southern Vietnam; to assess the utility potential of mNGS in terms of detecting a broad range of known/unknown pathogens in clinical samples, especially cerebrospinal fluid; and to estimate the economic burden attributed to paediatric encephalitis in southern Vietnam.

### Objectives

• To determine the epidemiology, clinical characteristics and hospital outcomes of paediatric encephalitis in southern Vietnam.
• To identify the frequency of infectious aetiologies and autoimmune causes detected in clinical samples, especially cerebrospinal fluid, of paediatric patients presenting with encephalitis allied with clinical outcome.
• To assess the utility potential of mNGS in the diagnosis of paediatric encephalitis in Vietnam.
• To estimate the economic burden attributed to encephalitis in children.

## Methods

### Study design and setting

The study will be prospectively conducted at Children’s Hospital 1 in Ho Chi Minh City in Vietnam between 2020 and 2022. Children’s Hospital 1 is a 1,600-bed hospital, and is the largest tertiary hospital for children coming from southern provinces of Vietnam with a catchment population of over 40 million. Annually, Children’s Hospital 1 has approximately 90,000 admissions. Of these, 150–200 cases presented with encephalitis, with infants and neonates accounting for ~35% ^11^.

### Participants

***Inclusion criteria.*** Any children admitted to the Department of Infectious Diseases and Neurology of Children’s Hospital 1 and fulfilling the case definition of encephalitis will be eligible to be invited to participate in the study.

***Exclusion criteria.*** Patients will be excluded if no informed consent was obtained.

### Case definition

Encephalitis in children ^1^ will be diagnosed when the patient has altered mental status (i.e., decreased or altered level of consciousness, lethargy, or personality change) lasting ≥24 hours with no alternative cause identified and ≥2 of these following criteria for a "possible" diagnosis or ≥3 of these following criteria for a "probable" diagnosis: documented fever ≥38°C (100.4°F) within 72 hours (before or after) presentation, generalized or partial seizures not fully attributable to pre-existing seizure disorder, new onset focal neurologic findings, cerebral spinal fluid white blood cell count ≥5 cells/mm ^3^, abnormality of brain parenchyma on neuroimaging suggestive of encephalitis that is new or appears to have acute onset, and abnormality on electroencephalogram that is consistent with encephalitis and not attributable to any other causes ^1^.

### Sample size

Given the descriptive nature of the present study, samples size calculation was not sought. We aim to carry out patient recruitment from 2020 to 2022 Based on our admission data, we anticipate that 100–150 patients will be enrolled every year.

### Data collection

Clinical data including dates of birth, admission and discharge, demographic data (date of birth, gender, place of residence, date admitted/discharged to/from the hospital), clinical signs, symptoms and syndromes, routine laboratory and imaging results (cerebrospinal fluid (CSF), urine and plasma samples, electroencephalogram (EEG), magnetic resonance imaging (MRI), computed tomography (CT) scan, diagnosis and differential diagnosis, associated diseases and co-morbidity, and treatment (immunotherapy with methylprednisolone, intravenous immunoglobulin, haloperidol, antiepileptic medications, tumour removal, plasmapheresis, etc.) will be collected.

Glasgow coma scale (GCS) and modified Rankin Scale (mRS) for children will used to measure the hospital outcomes of patients in this study. The mRS was previously used in a UK-based surveillance study of encephalitis in children, and in another study using data from Evelina Children’s Hospital, Great Ormond Street Children’s Hospital, St George’s Hospital, King’s College Hospital in London and Birmingham Children’s Hospital in Birmingham between 2007 and 2010 ^17, 21^.

We will also collect data on indirect- and direct- costs of illness from each study participant ^22^. Direct costs include costs attributable to medical health care and treatment during hospitalization. Indirect cost are costs incurred by parents or relatives who take care of the patients during hospitalization and seven days after discharge.

For laboratory analysis, we will collect a wide range of admission clinical samples including CSF, urine, plasma, rectal and throat swabs.

### Laboratory procedures

***Routine diagnostics.*** As part of routine care at Children’s Hospital 1, CSF specimens of patients presenting with brain infections are subject to cultured and/or examined by microscopy for detection of bacterial/fungal/ *Mycobacterium tuberculosis* infection with the use of standard methods when appropriate. Patients with encephalitis are further tested for herpes simplex virus and Japanese encephalitis virus using PCR and serology, respectively. Additional testing will include common causes of encephalitis such as enterovirus and varicella zoster virus, which will be carried out on archived CSF samples as part of the investigation of the present clinical study.

***Next-generation sequencing.*** Regardless of the results of investigation using classical assays outlined above, collected clinical samples (CSF, urine, plasma, and rectal and throat swabs) will be analysed using our in-house metagenomic next-generation sequencing (mNGS) workflow ^23– 25^. Biological samples will be sequenced and the obtained results will be compared against that of current (routine) diagnostic assays (especially PCRs). The aim is also to explore the utility of CSF and non-CSF samples for the diagnosis of encephalitis using mNGS. When appropriate, mNGS results obtained during the study period will be reported back to the treating physician only for their reference. This mNGS is currently not a standard diagnostic assay for encephalitis.

***Immunological assays.*** Undiagnosed patients with clinical presentations compatible with autoimmune encephalitis will then be tested for possible forms of the disease using EUROIMMUN assay (NMDAR) ^26^ and in-house assays (AMPA, GABA and VGKC-associated proteins) ^27^, which have been developed by Dr Irani and his group in Oxford. More specifically, the study involves exporting cerebral spinal fluid to Nuffield Department of Clinical Neurosciences, University of Oxford, Oxford, United Kingdom (Dr Sarosh Irani’s laboratory) and storage there for the purpose of immunological assays mentioned above.

### Data management

All available data will be entered into an electronic database (CliRes) developed by the IT department of the Oxford University Clinical Research Unit. Only the named investigators or their designee(s) will have access to this information. All other investigators will be regularly updated and will be granted access to data when requested. Patients will not be identified by their names. The investigators are responsible for maintaining all study records. The investigators are responsible for the timeliness, completeness and accuracy of the information in the original dataset and the clinical data management system. Laboratory staff will record specimens, their aliquots, and their storage location. All necessary tools, instruction, and training will be provided to all site staff involved in data entry to ensure the correct and consistent completion database prior to the study starting.

### Data storage

The investigators are responsible for retaining all essential data for at least five years after the completion of the study. Original paper documents will be maintained for a minimum of five years and electronic documents retained thereafter. All stored records are to be kept secure and confidential.

### Quality control procedures

The study will be conducted in compliance with the current approved protocol, International Conference on Harmonisation Good Clinical Practice (ICH GCP), relevant regulations and standard operating procedures. Regular monitoring will be performed according to ICH GCP. Data, samples and procedures will be evaluated for compliance with the protocol, standard operating procedures, regulatory requirements and terms of ethical approval. Records will be verified for accuracy against source documents and physical inventory of samples. The diagnostic laboratory at Children’s Hospital 1 conducts regular internal and external quality control procedures.

### Data analysis

Descriptive statistics will be employed to compare the epidemiology, clinical presentation, laboratory findings, patient treatment, and outcomes of patients. Data will be presented in the form of tables and charts for descriptive variables. When relevant, sophisticated statistical modelling approaches will be utilized to identify prognostic factors for (long-term) clinical outcomes. Association between pathogenic profile and outcomes of patients will be determined by univariable and multivariable analysis using logistic regression. All statistical analysis will be performed using Stata version 14 (StataCorp LP, College Station, TX, USA) and R; and p-values of ≤0.05 will be considered significant. We will estimate direct and indirect costs attributed to encephalitis using data collected from patients enrolled into the clinical studies.

### Consent

Written informed consent will be obtained from all study participants or their representatives before any data from patients is collected for the study. The study staff will discuss the study with the parent/representative of potential participants. Study staff will describe the purpose of the study, the study procedures, possible risks/benefits, the rights and responsibilities of patient, and alternatives to enrolment. The parent/representative will be invited to ask questions which will be answered by study staff, and they will be provided with appropriate numbers to contact if they have any questions subsequently. If the parent/representative agrees for their child to participate, they will be asked to sign and date two copies of an informed consent form ^28^. A copy of the form will be given to them to keep. If required, the parent/representative will be given up to 48 hours to consider for their children to take part in the study. In addition to the procedures above, illiterate signatories will have the informed consent form read to them in the presence of a witness who will sign to confirm that the form was read accurately and that the participant or representative agrees to participation. All informed consent forms will be written in the local language and will use terms that are easily understandable. 

### Ethics

This protocol and the relevant supporting documents have been approved by the ethical committee of Children’s Hospital 1 (1503/QD-BVND1) and the Oxford Tropical Research Ethics Committee (OxTREC 7-20) ^28^. The collection of all biological samples is performed as part of routine clinical assessments and are consistent with the local standard of care and good clinical practice.

### Dissemination and data sharing

The contributions of investigators from collaborating sites (Children’s Hospital 1, the Oxford University Clinical Research Unit, and the University of Oxford) will be recognized in authorship allocation in accordance with the guidelines of the International Committee of Medical Journal Editors. Data from this study is of substantial interest to the scientific and clinical research communities. Manuscripts arising from this study will, wherever possible, be submitted to peer-reviewed journals. All publications will acknowledge the study's funding sources. Data exchange complies with Information Governance and Data Security Policies in all of the relevant countries.

### Study status

At the time of protocol submission, all participating sites had been identified and recruitment had started.

## Discussion

The strength of our study is that it will contribute to the knowledge of scientists and clinicians in a low- and middle-income setting as currently, very little is known about the causative agents and the autoimmune features of encephalitis in children in Vietnam. Consequently, the clinical, microbiological, and genomic data produced by this study will be essential information for clinical practice and the development of future guidelines. This study will be conducted at the largest tertiary paediatric hospital in southern Vietnam. In this study, we will integrate the clinical assessments with microbiology, detailed genomics data, and immunological studies to further characterize the features of encephalitis in our setting.

Our study has several limitations. Firstly, ethical issues will complicate the way we conduct research in severely ill children with encephalitis, because children who are older than 12 years-old need to agree to join the study and sign a separate informed consent form on their own, which they cannot complete if the condition of their disease is severe. Secondly, there are no systematic guidelines ensuring accurate management of encephalitis (especially autoimmune encephalitis) at Children’s Hospital 1. Finally, data collection at a single paediatric hospital may limit the applicability of the results to other places in Vietnam.

Encephalitis is an obvious cause of morbidity and mortality of children in low- and middle-income countries including Vietnam. Generally, the main issues of encephalitis in children are the requirement for state-of-the-art laboratory tools for better and early diagnosis and the need for effective treatment. If this study shows that the metagenomics approach is useful, future research should focus on validity and applicability of proteomics-based experiments, molecular techniques or better markers of inflammation in order to develop rapid diagnostic tests for early detection of encephalitis, its pathogenic organisms, and methods to treat this condition effectively. With highly sensitive and specific diagnostic tools, the treatment of encephalitis in children would be significantly changed so that medical therapy could be safely and comprehensively administered or withheld. All of these factors may lead to costs reduction and the improvement of overall outcomes of paediatric encephalitis.

## Data availability

### Underlying data

No data are associated with this article.

### Extended data

Zenodo: Study protocol: The clinical features, epidemiology, and causes of pediatric encephalitis in southern_ICF_OxTREC APPROVAL. https://doi.org/10.5281/zenodo.4780771 ^28^.

This project contains the following extended data:

- INFORMED CONSENT FORM_Paediatric Encephalitis in Vietnam (01).docx
- INFORMED CONSENT FORM_Paediatric Encephalitis in Vietnam (02).docx
- OxTREC APPROVAL_Paediatric Encephalitis in Vietnam.pdf

Data are available under the terms of the Creative Commons Attribution 4.0 International license (CC-BY 4.0).
