# Supplementary material for: Study protocol: The clinical features, epidemiology, and causes of paediatric encephalitis in southern Vietnam
Source: Wellcome Open Res. 2022 Sep 13;6:133. Originally published 2021 May 28. [Version 3] doi: 10.12688/wellcomeopenres.16770.3 (PMC9579742; doi:10.12688/wellcomeopenres.16770.3)
Supplement: Peer review history

# Peer review: Reviewer response for version 3

**DOI:** 10.21956/wellcomeopenres.20170.r52364

**Published:** 2022-10-18

**Authors:** Emma C. Thomson

**Affiliations:** 1 MRC Centre for Virus Research, University of Glasgow, Glasgow, UK

The authors have addressed my previous comments very clearly.

Is the study design appropriate for the research question?

Yes

Is the rationale for, and objectives of, the study clearly described?

Yes

Are sufficient details of the methods provided to allow replication by others?

Partly

Are the datasets clearly presented in a useable and accessible format?

Not applicable

Reviewer Expertise:

Next generation sequencing, Virology, Clinical Infectious Diseases

I confirm that I have read this submission and believe that I have an appropriate level of expertise to confirm that it is of an acceptable scientific standard.

# Peer review: Reviewer response for version 2

**DOI:** 10.21956/wellcomeopenres.18800.r45722

**Published:** 2021-10-11

**Authors:** Emma C. Thomson

**Affiliations:** 1 MRC Centre for Virus Research, University of Glasgow, Glasgow, UK

This is an important and likely fruitful study investigating the causes of paediatric encephalitis in Vietnam. The investigators are experienced in the methods they plan to use to diagnose pathogens associated with encephalitis.

**Major Suggestions:**

 One more major criticism is that the investigators have not included negative controls and this will be really important for ascertaining whether or not pathogens that they find in the cases are unique to those cases or not. I think they should plan to sequence a certain number of control CSF samples taken for other reasons in order to have an idea about the background prevalence. They will inevitably find a lot of viral, bacterial and fungal derived RNA/DNA in their analysis - the question will be all about whether or not these are passengers or pathogens once the data have been collected.

**Minor issues:**

 I think that it would be useful to specify the timeframe following the onset of illness for collecting samples and have a minimum percentage of those collected during acute illness as often viral pathogens have quite transient viraemia; I'd perhaps aim for at least 50% with an acute presentation.

 The bioinformatic methods for the analysis have not been described in any detail - I think this would be helpful to see.

**For consideration only (may or may not be appropriate in the local context):**

 Another consideration which could be challenging would be to consider using brain biopsy material following limited post-mortem in the event of death with appropriate ethical approvals and permissions of next of kin.This would also facilitate ISH or similar to look for evidence of pathogens in the tissue.  Also, is it not possible to ask next of kin for written permission to carry out enhanced diagnostics in the case of low GCS if the patient does not have capacity?

**Summary:**

 In summary, the study is an important one in an area that needs research. I'd rethink the controls in particular and be explicit about the bioinformatic pipelines to be used in the analysis.

Is the study design appropriate for the research question?

Yes

Is the rationale for, and objectives of, the study clearly described?

Yes

Are sufficient details of the methods provided to allow replication by others?

Partly

Are the datasets clearly presented in a useable and accessible format?

Not applicable

Reviewer Expertise:

Next generation sequencing, Virology, Clinical Infectious Diseases

I confirm that I have read this submission and believe that I have an appropriate level of expertise to confirm that it is of an acceptable scientific standard, however I have significant reservations, as outlined above.

# Response

**Published:** 2022-08-06

**Authors:** Huong Nguyen

**Affiliations:** Oxford University Clincal Research Unit , Vietnam

**Reviewer** **: Emma C. Thomson**

 This is an important and likely fruitful study investigating the causes of paediatric encephalitis in Vietnam. The investigators are experienced in the methods they plan to use to diagnose pathogens associated with encephalitis.

**Major Suggestions:**

 One more major criticism is that the investigators have not included negative controls and this will be really important for ascertaining whether or not pathogens that they find in the cases are unique to those cases or not. I think they should plan to sequence a certain number of control CSF samples taken for other reasons in order to have an idea about the background prevalence. They will inevitably find a lot of viral, bacterial and fungal derived RNA/DNA in their analysis - the question will be all about whether or not these are passengers or pathogens once the data have been collected.

**Response:**

 We have added a sentence to elaborate on the inclusion of negative controls; line 145-150 reads “As negative controls, CSF samples from patients with laboratory-confirmed anti-NMDAR encephalitis and PCR-grade water will also be analysed. The obtained sequence data will be analysed to define the pathogen contents (including novel viruses) in the tested specimens using CZID (czid.net). Any virus detected by metagenomics will then be confirmed using specific PCR of the corresponding viruses or the detected viral sequences. Only those confirmed by PCR will be considered as a true positive”.

**Minor issues:**

 I think that it would be useful to specify the timeframe following the onset of illness for collecting samples and have a minimum percentage of those collected during acute illness as often viral pathogens have quite transient viraemia; I'd perhaps aim for at least 50% with an acute presentation.

**Response:**

 We have added some text to the manuscript to elaborate on this; line 109-113 reads “As per our routine practice, clinical samples are obtained within 12 hours from patients fulfilling the diagnostic criteria of possible or probable encephalitis. To maximise the chance of enrolling patients into the study during the acute illness phase, we will conduct the recruitment as soon as a patient with presumed encephalitis is identified. We will aim for at least 50% of those collected samples during acute illness”.

**The bioinformatic methods for the analysis have not been described in any detail - I think this would be helpful to see**.

**Response:**

 We have added some text to the manuscript to elaborate on this; line 146-150 reads “The obtained sequence data will be analysed to define the pathogen contents (including novel viruses) in the tested specimens using CZID (czid.net). Any virus detected by metagenomics will then be confirmed using specific PCR of the corresponding viruses or the detected viral sequences. Only those confirmed by PCR will be considered as a true positive”.

**For consideration only (may or may not be appropriate in the local context):**

 Another consideration which could be challenging would be to consider using brain biopsy material following limited post-mortem in the event of death with appropriate ethical approvals and permissions of next of kin. This would also facilitate ISH or similar to look for evidence of pathogens in the tissue.  Also, is it not possible to ask next of kin for written permission to carry out enhanced diagnostics in the case of low GCS if the patient does not have capacity?

**Response:**

 We have added some text to the manuscript to elaborate on this; line 108-109 reads “Obtaining brain biopsy is not allowed as part of routine care in our setting”.

**Reviewer** **: Elizabeth Molyneux**

 Neurological infections cause significant long-term morbidity and a high mortality rate. In children, most of the research, especially in LMICs has focussed on bacterial infections. This is because the tools for diagnosing common bacterial agents are widely available and treatment is known. These studies have fuelled the development and rollout of antibacterial vaccines with a large drop in paediatric cases: the picture is less rosy in neonates.

 With the reduction in bacterial neurological infections, the proportion of viral cases has increased. New diagnostics have made it simpler to identify the cause and the increase in antiviral medications has improved outcome. Now new diagnostic tools, such as metagenomic next generation sequencing (mNGS) have entered the field that allow a wider net to be cast in trying to identify the aetiological agents. Also, there is new and heightened interest in autoimmune causes of encephalitis which are probably responsible for a significant number of the present day undiagnosed causes of encephalitis.

 In Vietnam, an aetiological study of encephalitis was carried out in 2003-2004, and 41% were of viral cause, Japanese encephalitis (26%) enteroviruses (9.3 %) and dengue (4.6%) being the most common; 54% did badly of whom 29% died. Since then, different, sometimes novel viruses have been identified as causing encephalitis.

 In this descriptive study, the investigators plan to collect detailed clinical, laboratory, imaging and outcome data on children admitted to one large Vietnamese children’s hospital in 2021-2022. They anticipate that 100-150 children will be enrolled. A wide range of clinical samples will be taken on admission on which microscopy and culture will be undertaken in real time according to the hospital routine standard of care, for bacteria, fungi and TB, and if appropriate, serology and PCR for Japanese encephalitis and herpes simplex viruses. Stored samples will be tested for varicella zoster virus and enteroviruses. All CSF, plasma, throat, urine and stool samples will undergo mNGS regardless of the results from other diagnostic tests. On the samples in which no causative agent has been identified immunological assays will be done in Oxford using EUROIMMUN assay (NMDAR) and in-house assays (AMPA, GABA and VGKC-associated proteins).

**I only have one or two questions to ask, namely;**

**What age range of children is to be enrolled and will results be analysed by age?**

**Response:**

 We have added some text to the manuscript to elaborate on this; line 80-82 reads “Any children age less than or equal to 16 years admitted to the Department of Infectious Diseases and Neurology of Children’s Hospital 1 and fulfilling the case definition of encephalitis will be eligible to be invited to participate in the study”.

**What does the routine immunisation programme for children include and at what ages? I assume this data will be collected.**

**Response:**

 We have added some text to the manuscript to elaborate on this; line 114-120 reads “Our local context has a national immunisation programme for children (less than or equal to 16 years of age) and this data will also be collected. The pathogens and diseases that can be covered by our national immunisation programme for children include: tuberculosis, hepatitis B virus, diphtheria, tetanus, pertussis, poliomyelitis, HiB diseases, rotavirus, pneumococcal disease, meningococcal disease caused by serogroups B and C, influenza, measles, meningococcal ACWY, Japanese encephalitis, measles, mumps, rubella, varicella, hepatitis A, typhoid, and cholera”.

**Will nutritional status be recorded?**

**Response:**

 Yes, nutritional status will be recorded (line 103).

**The study is to be undertaken at a large tertiary hospital. How do children get admitted to the hospital? Are most of them referred from other health facilities or do most self-refer?**

**Response:**

 We have added some text to the manuscript to elaborate on this; line 75-76 reads “Children are either referred to our hospital directly from home or from other health facilities”.

**How long have children been unwell before being admitted and what previous treatment are they likely to have received? (All of which I presume will be collected) Will this affect any of the anticipated diagnostic tests.**

**Response:**

 We have added some text to the manuscript to elaborate on this; line 109-113 reads "As per our routine practice, clinical samples are obtained within 12 hours from patients fulfilling the diagnostic criteria of possible or probable encephalitis. To maximise the chance of enrolling patients into the study during the acute illness phase, we will conduct the recruitment as soon as a patient with presumed encephalitis is identified. We will aim for at least 50% of those collected samples during acute illness”. The vaccination status, history of illness, prior medications given will also be recorded (line 101-102).

# Peer review: Reviewer response for version 1

**DOI:** 10.21956/wellcomeopenres.18495.r44137

**Published:** 2021-06-07

**Authors:** Elizabeth Molyneux

**Affiliations:** 1 Department of Paediatrics, University of Malawi, Blantyre, Malawi

Neurological infections cause significant long-term morbidity and a high mortality rate. In children, most of the research, especially in LMICs has focussed on bacterial infections. This is because the tools for diagnosing common bacterial agents are widely available and treatment is known. These studies have fuelled the development and rollout of antibacterial vaccines with a large drop in paediatric cases: the picture is less rosy in neonates.

 With the reduction in bacterial neurological infections, the proportion of viral cases has increased. New diagnostics have made it simpler to identify the cause and the increase in antiviral medications has improved outcome. Now new diagnostic tools, such as metagenomic next generation sequencing (mNGS) have entered the field that allow a wider net to be cast in trying to identify the aetiological agents. Also there is new and heightened interest in autoimmune causes of encephalitis which are probably responsible for a significant number of the present day undiagnosed causes of encephalitis.

 In Vietnam, an aetiological study of encephalitis was carried out in 2003-2004, and 41% were of viral cause, Japanese encephalitis (26%) enteroviruses (9.3 %) and dengue (4.6%) being the most common; 54% did badly of whom 29% died. Since then different, sometimes novel viruses have been identified as causing encephalitis.

 In this descriptive study, the investigators plan to collect detailed clinical, laboratory, imaging and outcome data on children admitted to one large Vietnamese children’s hospital in 2021-2022. They anticipate that 100-150 children will be enrolled. A wide range of clinical samples will be taken on admission on which microscopy and culture will be undertaken in real time according to the hospital routine standard of care, for bacteria, fungi and TB, and if appropriate, serology and PCR for Japanese encephalitis and herpes simplex viruses. Stored samples will be tested for varicella zoster virus and enteroviruses. All CSF, plasma, throat, urine and stool samples will undergo mNGS regardless of the results from other diagnostic tests. On the samples in which no causative agent has been identified immunological assays will be done in Oxford using EUROIMMUN assay (NMDAR) and in-house assays (AMPA, GABA and VGKC-associated proteins).

 I only have one or two questions to ask, namely;

- What age range of children is to be enrolled and will results be analysed by age?
- What does the routine immunisation programme for children include and at what ages? I assume this data will be collected.
- Will nutritional status be recorded?
- The study is to be undertaken at a large tertiary hospital. How do children get admitted to the hospital? Are most of them referred from other health facilities or do most self-refer?
- How long have children been unwell before being admitted and what previous treatment are they likely to have received? (All of which I presume will be collected) Will this affect any of the anticipated diagnostic tests.

The investigators are to be congratulated for proposing a careful, all-round descriptive study of an important topic with the real and practical possibility of improving diagnosis, treatment and outcome for children with encephalitis.

Is the study design appropriate for the research question?

Yes

Is the rationale for, and objectives of, the study clearly described?

Yes

Are sufficient details of the methods provided to allow replication by others?

Yes

Are the datasets clearly presented in a useable and accessible format?

Not applicable

Reviewer Expertise:

My research has been in paediatric bacterial meningitis and into managing convulsions in a low income country.

I confirm that I have read this submission and believe that I have an appropriate level of expertise to confirm that it is of an acceptable scientific standard.
